# Gd-EOB-DTPA-Enhanced MR Guidance in Thermal Ablation of Liver Malignancies

**DOI:** 10.1371/journal.pone.0109217

**Published:** 2014-12-26

**Authors:** Christian Rosenberg, Andrea Jahn, Tilman Pickartz, Ulrich Wahnschaffe, Maciej Patrzyk, Norbert Hosten

**Affiliations:** 1 Institute of Diagnostic Radiology and Neuroradiology, University Medicine Greifswald, Greifswald, Mecklenburg-Vorpommern, Germany; 2 Clinic of Internal Medicine, Division of Gastroenterology, Endocrinology and Nutritive Medicine, University Medicine Greifswald, Greifswald, Mecklenburg-Vorpommern, Germany; 3 Clinic of General Surgery, Visceral, Thoracic and Vascular Surgery, University Medicine Greifswald, Greifswald, Mecklenburg-Vorpommern, Germany; West German Cancer Center, Germany

## Abstract

**Objective:**

To evaluate the potency of Gd-EOB-DTPA to support hepatic catheter placement in laser ablation procedures by quantifying time-dependent delineation effects for instrumentation and target tumor within liver parenchyma. Monitoring potential influence on online MR thermometry during the ablation procedure is a secondary aim.

**Materials and Methods:**

30 cases of MR-guided laser ablation were performed after i.v. bolus injection of gadoxetic acid (0.025 mmol/Kg Gd-EOB-DTPA; Bayer Healthcare, Berlin, Germany). T_1_-weighted GRE sequences were used for applicator guidance (FLASH 3D) in the catheter placement phase and for therapy monitoring (FLASH 2D) in the therapy phase. SNR and consecutive CNR values were measured for elements of interest plotted over time both for catheter placement and therapy phase and compared with a non-contrast control group of 19 earlier cases. Statistical analysis was realized using the paired Wilcoxon test.

**Results:**

Sustainable signal elevation of liver parenchyma in the contrast-enhanced group was sufficient to silhouette both target tumor and applicator against the liver. Differences in time dependent CNR alteration were highly significant between contrast-enhanced and non-contrast interventions for parenchyma and target on the one hand (p = 0.020) and parenchyma and instrument on the other hand (p = 0.002). Effects lasted for the whole procedure (monitoring up to 60 min) and were specific for the contrast-enhanced group. Contrasting maxima were seen after median 30 (applicator) and 38 (tumor) minutes, in the potential core time of a multineedle procedure. Contrast influence on T1 thermometry for real-time monitoring of thermal impact was not significant (p = 0.068–0.715).

**Conclusion:**

Results strongly support anticipated promotive effects of Gd-EOB-DTPA for MR-guided percutaneous liver interventions by proving and quantifying the delineating effects for therapy-relevant elements in the procedure. Time benefit, cost effectiveness and oncologic outcome of the described beneficiary effects will have to be part of further investigations.

## Introduction

Image-guided tumor ablation in the liver has become an accepted high-end technical approach in multimodal cancer therapy [1]–[5]. As a mainly palliative procedure with a perceived curative potential it benefited from latest technical developments, such as MR guidance and impartial MR thermometry for thermal ablation procedures [6]–[11]. Meeting the requirements of modern oncology concepts, while still facing a diverse technical pattern, demands specially trained personnel [12]. Oncologic quality measures under these circumstances are therefore still highly dependent on the interventionalist's experience. At the same time MR-guided tumor ablation is a labor-intensive and time-consuming procedure, which is especially relevant when it comes to profitable practice in outpatient centers. With respect to both criteria, profitability and quality, a great demand for standardization on the one hand and simplification on the other hand has been noticed not only in the community of interventional radiologists [13]. Standardization and simplification both also promote procedure automating needs currently addressed by the healthcare industry.

Laser-induced thermal ablation in combination with MR guidance is recognized as the most efficient technical setting for online therapy monitoring within the hepatic target zone [11], [14], [15]. High-resolution thermometry of fiber and target tumor, as well as fiber placement in the liver benefit from MR-specific imaging qualities. MRI performance is superior to CT in displaying non-enhanced focal lesions [16], which is important, as the procedure – just as any other image-guided liver intervention – is lasting too long to maintain a solitary extracellular intravenous (i.v.) contrasting of the target lesion. Catheter placement in general benefits from multiplane reconstructive imaging [17]. The importance of modern liver-specific intracellular contrast media, such as Gd-EOB-DTPA, is widely respected and has made MRI the imaging gold standard in staging focal hepatic disease [18]–[23]. With a broader availability, these contrast agents qualify for periinterventional liver imaging, especially in metastatic disease. Gadolinium-based lasting signal enhancement in hepatic tissue at late-phase is a substance- and MR-specific property [23]. With a given metallic catheter mandrin and a non-enhancing target tumor contrast-induced elevation of the parenchymal signal is anticipated to promote the feasibility of catheter placement. Single reports describe beneficiary effects when using intracellular contrast media in guided interventions [24], [25]. So far, nobody has ever quantified these promotive effects, nor tested their reliability.

The aim of this study was to retrospectively quantify time-dependent contrasting effects between catheter mandrin and liver parenchyma on the one hand and between target lesion and surrounding liver parenchyma on the other hand when using intracellular contrast imaging to support hepatic catheter placement in laser ablation procedures. It also intended to rule out contrast-induced disturbing side effects on the performance of online MR thermometry during the ablation procedure.

## Materials and Methods

### Study design and patient population

49 MR-guided ablation procedures in 26 patients were chronologically chosen from the population of an ongoing surveillance study. The study protocol, entitled “Use of GD-EOB-DTPA in MR-guided thermal ablation of liver tumors”, was approved by the institutional ethics committee (registration number BB 93/08a, Ethics Committee of the Ernst Moritz Arndt University Greifswald). Written informed consent was obtained from all patients at least 1 day prior to treatment. Within the 49 procedures 19 were pre-existent unenhanced image-guided interventions and served as a control group (G2). The other 30 procedures comprised i.v. bolus application of Gd-EOB-DTPA (G1) before catheter placement.

Patients (18 male and 8 female, median age 62 years, range 37–83 years) were regularly admitted for MR-guided laser ablations. 17 patients were treated in 1 session, 9 patients (7 male and 2 female) received more than one treatment session (two to seven per patient, median 3). 49 tumor treatments targeted 45 tumors: hepatocellular carcinomas in 6 cases, carcinoma metastases of colorectal (n = 27), breast (n = 3), endometrial (n = 3), neuroendocrine (n = 2), pancreatic (n = 1), squamous cell (n = 1), cholangiocellular (n = 1) origin and malignant melanoma metastasis in one case. Out of 45 tumors and based on the largest tumor diameter we treated 32 small (≤3 cm, Ø 1.74 cm; SD 0.60) and 13 intermediate tumors (3–5 cm; Ø 3.75 cm; SD 0.53). In 4 cases larger tumors with a diameter>5 cm (Ø 6.31 cm; SD 1.66) were treated with cytoreductive intent.

### MR-guided laser ablation

#### Contrast agent

Gadoxetic acid (Gd-EOB-DTPA; Bayer Healthcare, Berlin, Germany) is a bimodal MR-suitable contrast agent that combines the benefits of non-specific extracellular and liver cell-specific agents in one [26]. The T_1_-relaxivity measured in water is 4.2 mmol^−1^s^−1^ at 1.5 T and increases in human blood to 7.3 mmol^−1^s^−1^ at 37°C. Even though administered in smaller dosages compared to similar extracellular derivatives (0.1 vs. 0.025 mmol/kg) the substance is characterized by an early unspecific dynamic vascular allocation followed by rapid, sufficient liver enhancement because of specific liver cell uptake. As a linear ionic gadolinium chelate it offers a high thermodynamic stability (23.5 log K_therm_ pH 14 or 18.7 log K_cond_ pH 7.4, respectively) [26], [27].

#### Therapeutic procecure

Procedures were identical in both groups. They were fully performed in the MRI suite using a closed 1.5 T MR scanner with flexible spine/body array coils and in-room console (Siemens Healthcare, Erlangen, Germany) according to an established protocol [14], [17]. The same experienced (7 years) interventional radiologist performed all 49 interventions.

Breath-hold T1-weighted GRE FLASH 3D sequences (FOV 300; TR 4.8; TE 2.2; SD 3 mm; FA 10°) were used to guide applicator placements (average 2 applicators per target, range 1–4). A total of 99 applicators were placed in 49 procedures. Gadoxetic acid was applied (i.v. bolus injection of 0.025 mmol/kg body weight prior to the intervention) in 30 cases to promote placement of 67 applicators. The laser applicator system (RoweMed, Parchim, Germany) consisted of a titanium needle with tetragonally sharpened tip (diameter 1.5 mm) and was surrounded by a heat-resistant teflon catheter (outer diameter 1.8 mm or 5.5 F). System lengths varied between 12 and 18 cm. Being properly positioned, the titanium needle was removed and replaced by a flexible laser fiber consisting of silica with a diameter of 1.1 mm and a diffusor tip of 30 mm length. An Nd:YAG laser generator (neodymium yttrium aluminum garnet laser; Dornier, Germering, Germany) operating at a wavelength of 1.064 nm and a 50-ml syringe pump (IVAC Medizintechnik GmbH Giessen, Germany) for cooling were placed outside the MRI suite.

#### T1 magnitude thermal monitoring

Heat distribution was visualized through repetitive acquisition of T1 FLASH 2D sequences (TE 4.8 ms, TR 100 ms, BW 260 Hz/pixel, flip angle 70°, slice thickness 5 mm, fat saturation). Ten to fifteen axial slices per acquisition were sufficient to cover the target region. A rise in tissue temperature increased the T1 relaxation time, resulting in a lower T1 signal [28]. Derived from earlier practice the margin of the signal loss qualitatively determined lethal impact and anticipated tissue necrosis [14].

### Image Analysis

For image analysis, a workstation (Siemens Healthcare, Erlangen, Germany) was used. Elliptic regions of interest (ROI) to measure signal intensity (SI) were manually placed in areas representing liver parenchyma, tumor tissue, metallic mandrin and thermal impact zone. Additional ROI placement was performed within background air outside the patient body for each slice to measure corresponding noise. Double measurements were averaged per slice and tissue quality. During catheter placement eight time-scattered random sequences were analyzed. ROI sizes depended on the availability of homogeneous tissue signal within a plane and varied between 1.1 cm^2^ in the liver (range 0.34–2.75; SD 0.3), 0.23 cm^2^ within tumor tissue (range 0.03–0.72; SD 0.17), 0.09 cm^2^ for applicator mandrins (range 0.02–0.19; SD 0.04) and 9.87 cm^2^ for noise determination (range 3.85–14.31; SD 1.69). Within the therapy phase at least 4 time points were chosen for ROI placement (liver 1.05 cm^2^ (range 0.4–2.43; SD 0.4), ablation zone 0.38 cm^2^ (range 0.06–0.74; SD 0.18) and noise 7.57 cm^2^ (range 3.68–12.39; SD 0.87)). ROI settings were copied from one to another slice within an individual therapy session but needed adjustment in selective cases to avoid inclusion of unappreciated anatomic structures or thermal impact.

### Statistical and graphical analysis

A total of 49 treatment sessions were analyzed retrospectively. Group 1 comprised signal measurements both during catheter placement and therapy phases in 30 procedures. Group 2 instead, due to retrospective image data availability, had to be divided into 15 out of 19 cases, which were found sufficient to evaluate the catheter placement phase and 6 out of 19 cases, which were elective for evaluation of the therapy phase. Consecutively, 2 out of 19 unenhanced procedures were eligible for both evaluation parts.

SPSS for windows (version 18.0; SPSS, Chicago, USA) and DIAdem (version 10.1 DIAdem; National Instruments, Austin, USA) were used for statistical and graphical evaluation. Arithmetic means of double measurements were calculated and used for further processing to define SNR and CNR. SNR (*SI_structure_/SD_noise_)* was calculated for liver parenchyma, target tumor and metallic mandrin of the applicator during catheter placement phase and for liver parenchyma and thermal impact zone only within the therapy phase. CNR (*SI_structure1_ – SI_structure2_/SD_noise_*) were calculated to evaluate the liver-to-lesion, liver-to-applicator, applicator-to-lesion and liver-to-ablation zone contrasts. Standard deviation and mean values were calculated in all settings. Time points of sequential imaging, both for catheter placement and therapy monitoring phase, were assigned to 5-minute intervals each. The graphical work-up over time shows delta values to achieve intraindividual comparability. Curves are shown as a compensating 4^th^ grade polynomial function due to small case numbers in the therapy phase for group 2 or as 5^th^ grade polynomial functions for all other cases [29], [30]. Differences of resulting numerical series were tested for statistical significance between the two main groups, contrast versus non-contrast, on the one hand and enhancing versus non-enhancing tumors on the other hand. Using the Wilcoxon test, the 95% confidence level was set to 0.05 to indicate statistically significant differences.

## Results

### Contrast-induced tissue signal alteration in the target organ

#### Liver

**Figure 1:**
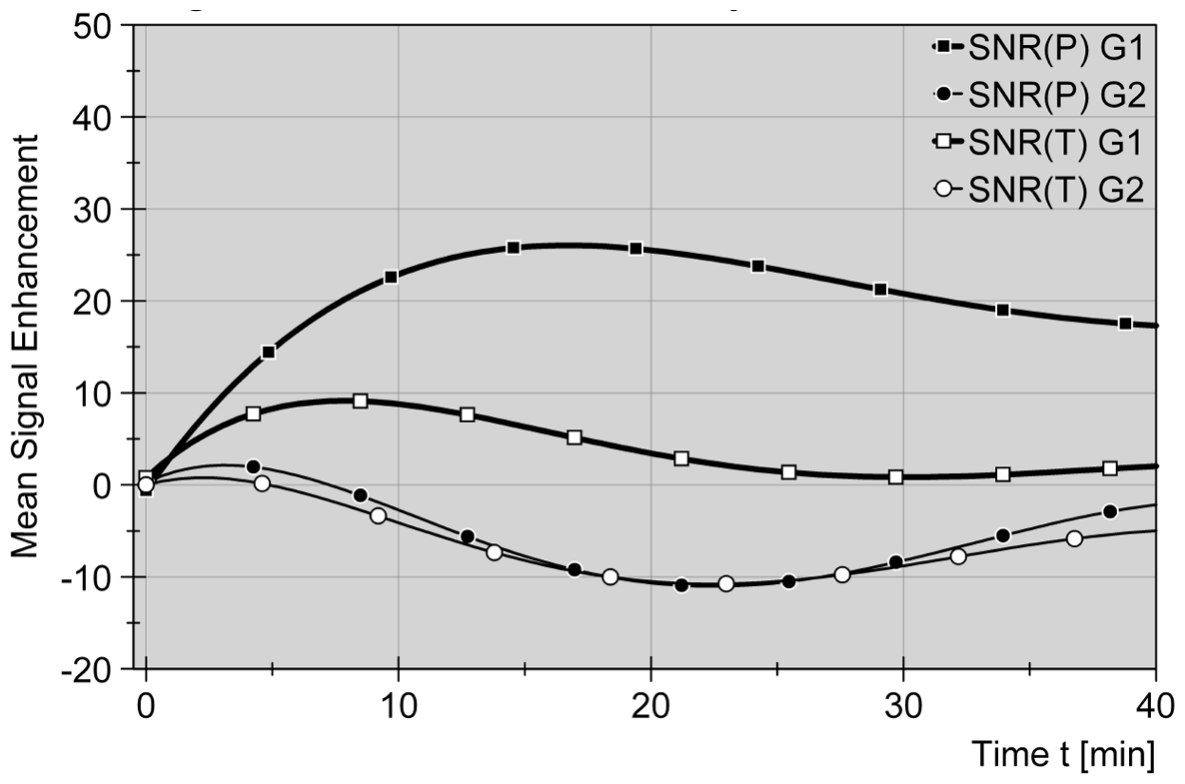
Comparative signal alteration for liver parenchyma and target tumors in group 1 (Gd-EOB-DTPA) and group 2 (non-contrast). Signal-to-noise ratios (SNR, delta values) are displayed as a function of time after bolus injection of Gd-EOB-DTPA (0.025 mmol/Kg i.v.) in group 1 and start time in group 2. Standardized curves are compensating 5^th^ grade polynomial functions. A rapid contrast uptake in group 1 (G1) leads to an enduring signal elevation in liver parenchyma (P) during catheter placement phase (–▪–). Values in group 2 (G2) are oscillating around zero as non-contrast signals are not significantly altered (–•–). Contrast-induced tumor (T) signal increase appears in the early extracellular phase (<10 minutes), while the curve (–□–) is approximating zero and non-contrast values (–○–) thereafter. As a consequence the graph displays a favorable gap between P^G1^ and T^G1^ on the one hand and P^G1^ and both P^G2^ + T^G2^ on the other hand.

After i.v. bolus injection of gadoxetic acid the liver parenchyma showed a significant homogeneous increase (SNR) in signal intensity on T1-weighted images during catheter placement phase (p = 0.002). (see fig. 1) Signal alteration increased gradually from pre-contrast images to maximum values after median 17.5 minutes (range 5–60 min, 50% after median 3.3 min), after 16 minutes according to the polynomial graph. Signal intensity approximated a steady state and remained constantly high for the rest of the catheter placement phase (monitoring up to 60 min).

#### Target tumor

Three of four hepatocellular carcinomas (all three from one individual who was treated in three sessions) in the contrast-enhanced group (G1) did not show any contrast enhancement at all after bolus application of Gd-EOB-DTPA. All other target tumors, comprising metastases of colorectal, pancreatic, breast and squamous cell carcinomas as well as one metastatic choroid melanoma, revealed contrast uptake in the early vascular phase after intravenous injection. Early phase SNR peaks were seen within the first 5 minutes after application for the majority of tumors (64%), between 5 and 10 minutes post injectionem (p.i.) in most of the other cases (total range 5–60 min, median 5 min, polynomial graph 7 min). Focal enhancement ranged between 7% and 170% (median 30%) for maximum peak, with the singular enhancing HCC representing the highest peak. Inhomogeneous intralesional contrast enhancement in the early phase was followed by a subsequent and constant decrease of signal intensity or SNR, as depicted by the compensating polynomial curve (see fig. 1). All individual SNR curves homogenously approximated a steady state with stable progression between 20 and 25 minutes after administration of gadoxetic acid. Relative SNR levels ranged between 0% and 25% of the pre-contrast amount still at 40 minutes p.i. (p = 0.017).

### Contrast-induced periprocedural imaging effects

#### Liver-to-tumor contrast

**Figure 2:**
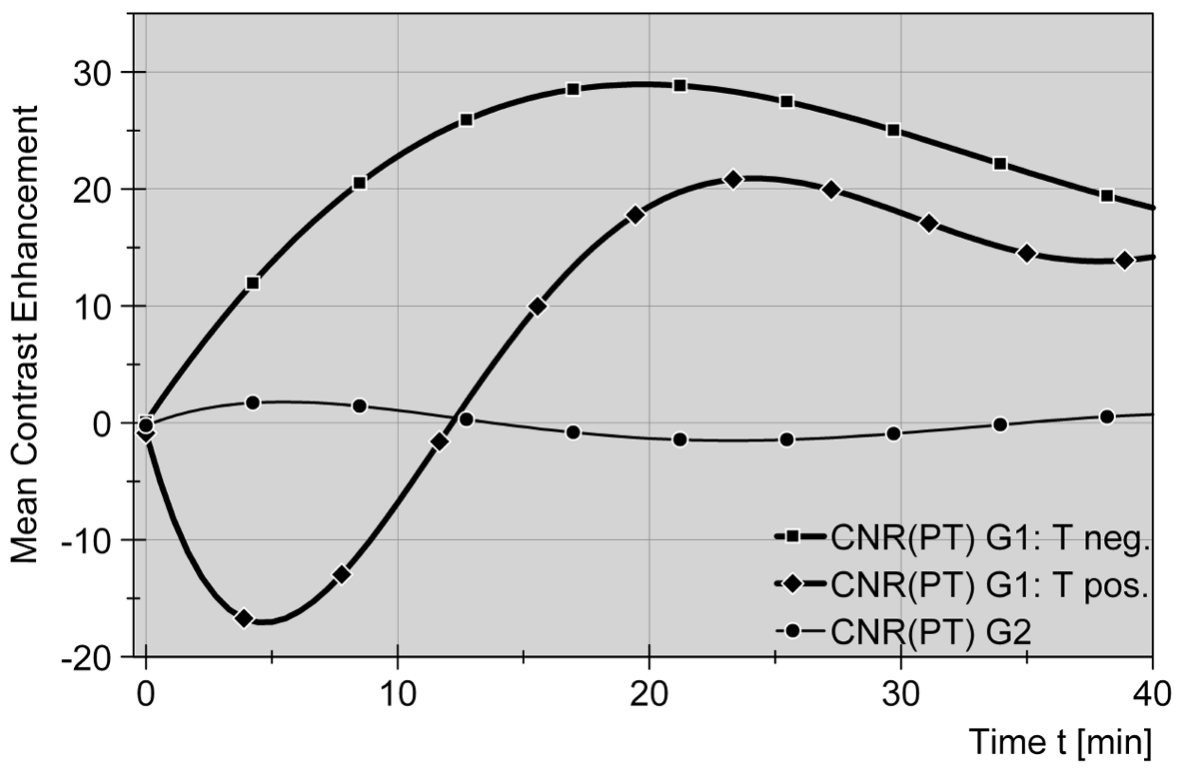
Liver-to-tumor contrast in dependence of Gd-EOB-DTPA application on the one hand and absence or presence of early phase tumor enhancement on the other hand. Contrast-to-noise ratios (CNR, delta values) are displayed in the same manor as SNR curves in figure 1. While sharing the favorable increase in contrast between parenchyma (P) and target tumor (T) at late phase, enhancing (Tpos) and non-enhancing (Tneg) tumors in group 1 (G1) show different curve progressions in the early phase after bolus contrast. Reversion from negative to positive delta CNR values (–♦–) and traversing an early equilibrium is characteristic for enhancing tumors (Tpos^G1^) in the given setting.

**Figure 3:**
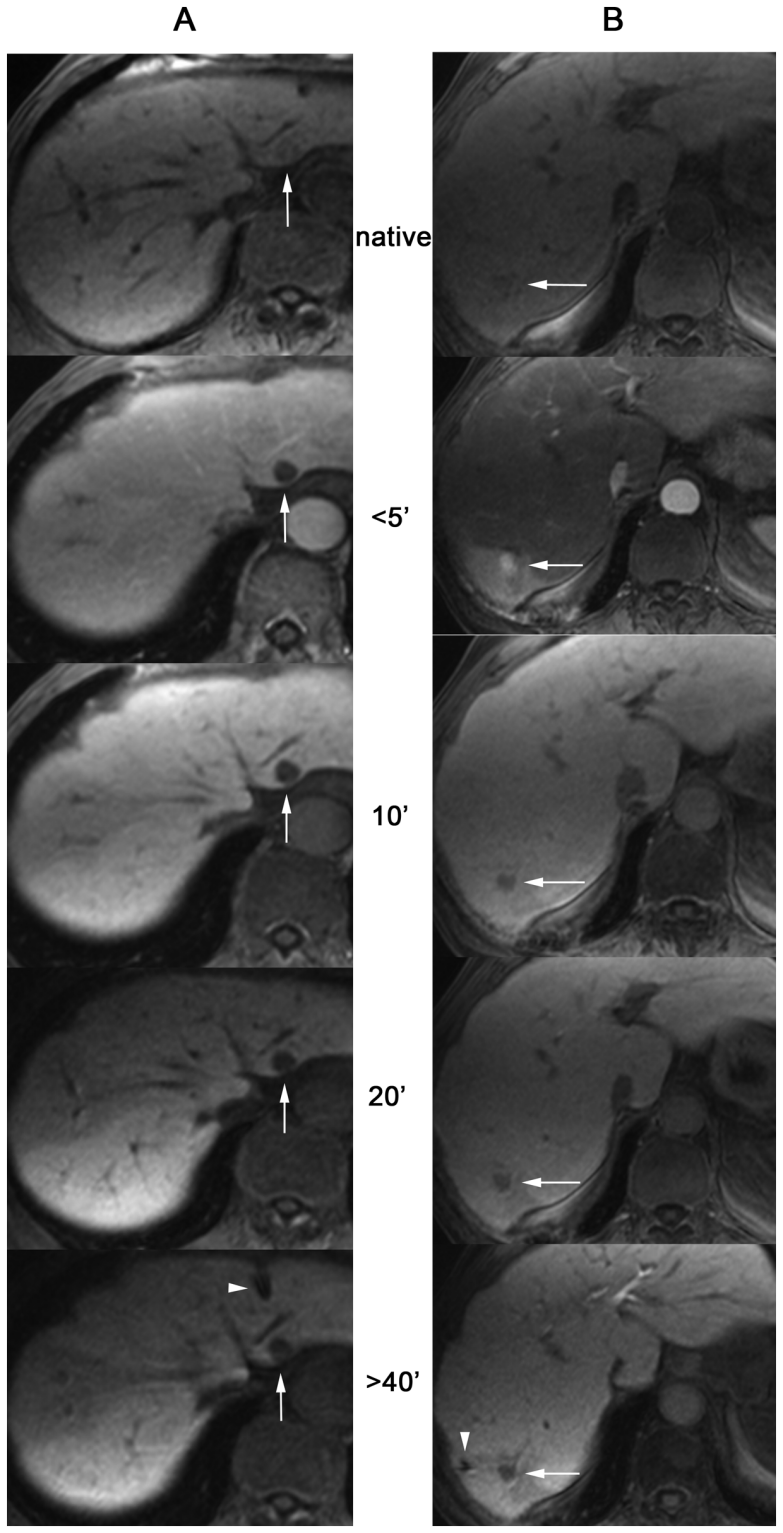
Liver-to-tumor contrast promotes planning and placement of applicators. Dynamic imaging (breath-hold T1-weighted GRE FLASH 3D sequences) of both non-enhancing Tneg^G1^ (A) and enhancing Tpos^G1^ (B) during planning and catheter placement phase in two different cases. Column A shows a sustainable high-low contrast between liver parenchyma and target tumor (arrow), whereas column B shows a transient low-high contrast between parenchyma and tumor (arrow). Advantageous target outlining at late phase initiates catheter (arrow heads) approach in both cases.

In monitoring gadolinium-induced contrasting effects between liver and target tumor in the early vascular phase, enhancing and non-enhancing tumors had to be distinguished, because their different properties resulted in opposite CNR curve progression within the first ten minutes after contrast administration (see fig. 2). A lack of tumor enhancement resulted in an early rapid increase of exclusively positive CNR values, representing a constant high-low signal contrast between liver and target tumor (see fig. 3). Maxima were reached at about 17 minutes after contrast application (50% after 8 min, both according to the polynomial graph, calculated: median 37.5 min and 12.2 min, respectively). Instead, in cases showing initial target tumor enhancement, CNR curve progression revealed negative values in the beginning with maxima between 1 and 5 minutes p.i., representing a low-high signal contrast between liver and target tumor. Passing an equilibrium phase the CNR curve progression changed to positive values between 12^th^ and 18^th^ minute p.i. reaching maxima after 20 to 30 minutes (see fig. 2). All CNR curves in common, enhancing and non-enhancing tumors of group one, revealed transition into near steady states with CNR values being inter-individually leveled. In the non-enhanced group SNR curves (delta values) for liver and tumor tissue were not significantly different over time, and therefore were not resulting in a beneficial tissue imaging contrast (CNR approximately +/−0). (see fig. 2) Differences in CNR levels were significant (p = 0.020) when being compared with unenhanced group two.

#### Liver-to-applicator contrast

**Figure 4:**
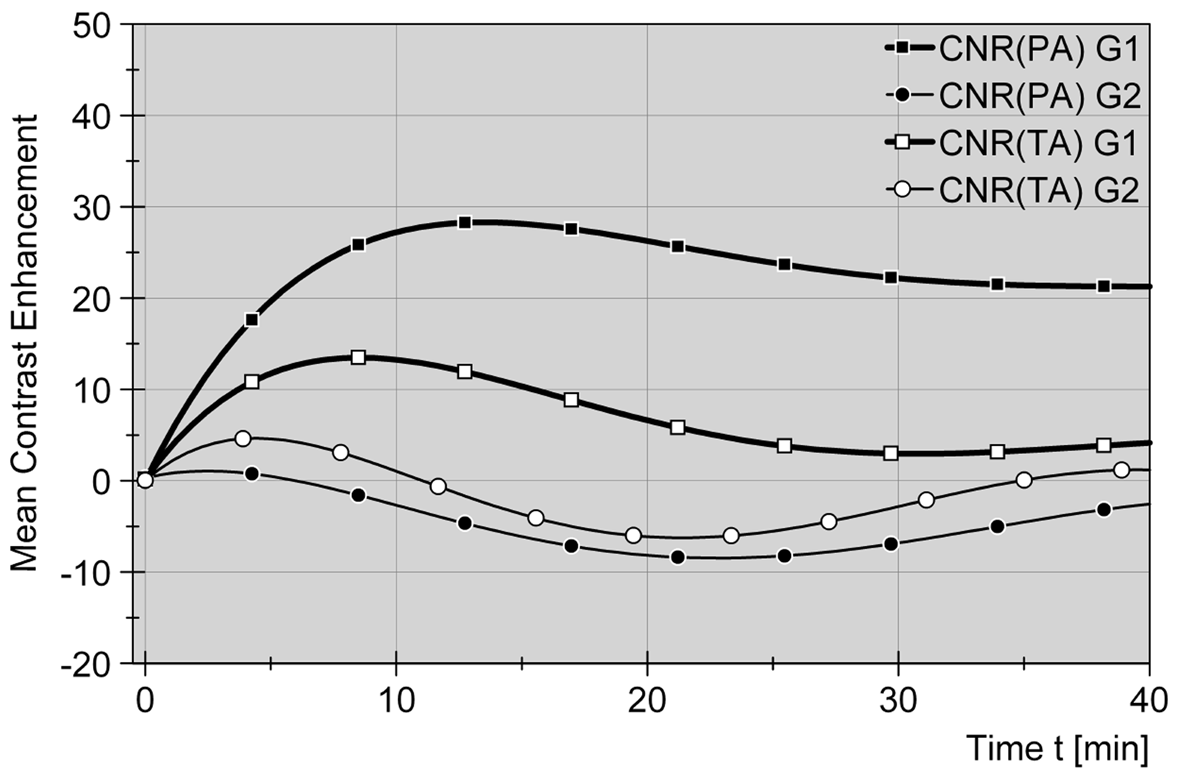
Tissue-to-instrument contrast alteration in dependence of Gd-EOB-DTPA application. The graph shows a superior potency of cellular Gd-EOB-DTPA uptake to silhouette a metallic applicator (A) against a parenchymal (P) background (–▪–), as compared with non-contrast image guiding (–•–) in group 2 (G2).

**Figure 5:**
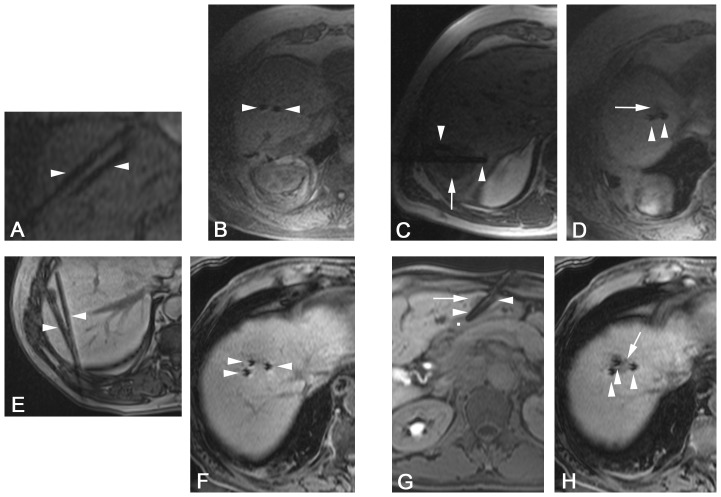
Liver-to-instrument contrast visualizes the procedure. Dynamic imaging (breath-hold T1-weighted GRE FLASH 3D sequences) confirms the advantageous properties of late phase Gd-EOB-DTPA uptake showing cases of non-contrast (group 2, A-D) and contrast-enhanced guidance (group 1, E-H). Four different cases each, demonstrate multiple applicator (arrow heads) placement approaching different target tumors (arrows). Image G shows a fluid collection (▪) consistent with protective saline injection towards the stomach and prior to the ablation.

In group one, CNR values, representing a delta in signal intensity between treated liver and introduced applicator (including titanium mandrin during catheter placement phase), gradually increased from pre-contrast images to highest levels at 13.5 minutes after Gd-EOB-DTPA administration, half the way at 3.1 minutes (calculated: 100% at median 30 min, 50% at median 3.2 min). (see fig. 4) It outlasted the remaining catheter placement phase (see fig. 5). The elevating effect on tissue signal contrast in the enhanced group one was highly significant (p = 0.002) when being compared with the unenhanced group two.

#### Tumor-to-applicator contrast

Not unexpected, without supplying intravenous Gd-EOB-DTPA in control group two, the existing signal contrast between target tumor and applicator was not altered over time during the procedure. The same phenomenon could be found for non-enhancing tumors of group one (i.v. contrast). With a constantly low signal in the metal applicator (when being positioned), early uptake (SNR) in all other target tumors lead to an instant increase of CNR values showing maxima at 7.6 minutes, as depicted by the compensating polynomial curve (median 5 min, range 5–50 min). Thereafter a sufficient washout decreased CNR curve progression to merge into a near steady state during 25^th^ and 27^th^ minute p.i. Even at 40 minutes p.i. CNR values were still about 30% higher than at starting time of the procedure (p = 0.008).

### Contrast influence on therapy monitoring

#### Liver

Being differentiated from the catheter placement phase, the therapy phase started with switching-on laser power and lasted standard 20 minutes. As being presented in the above text SNR and CNR curves were in a steady state at that stadium of the procedure. SNR values remained stable throughout the therapy phase. Also, there was no significant difference in signal alteration (ΔSNR) comparing group one and group two at 5, 10, 15 and 20 minutes after starting the laser ablation (p_5_ = 0.068; p_10_ = 0.715; p_15_ = 0.173; p_20_ = 0.593).

#### Target zone

**Figure 6:**
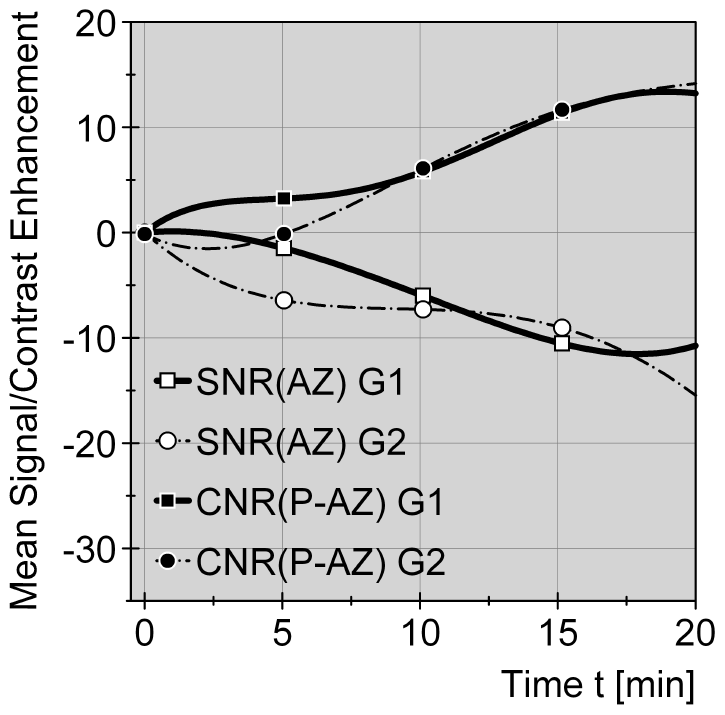
Comparative signal and contrast alterations for liver parenchyma and thermal impact zone in group 1 (Gd-EOB-DTPA) and group 2 (non-contrast) during therapy phase. Ratios (SNR+CNR, delta values) are displayed as a function of time after technically starting an ablation cycle in group 1 (Gd-EOB-DTPA) and group 2 (non-contrast). Standardized curves are compensating 4^th^ grade polynomial functions. Graphs show homogeneous decrease of SNR values within the thermal impact or ablation zones (AZ), consistent with temperature-induced T1 signal drop, in both groups 1+2 (G1, G2). At the same time CNR values (parenchyma P vs. AZ) increase for both groups in a parallel manor. It seams, thermometry monitoring is not influenced by Gd-EOB-DTPA application.

**Figure 7:**
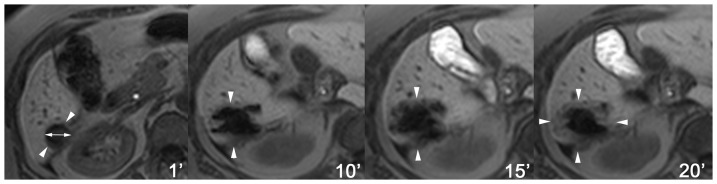
MR thermometry at late phase contrast-enhanced imaging. Thermometric MR imaging (T1 FLASH 2D) delineates the development of a T1-hypointense zone of thermal impact (arrowheads show boundaries) encountering a 2 cm hepatic metastasis (double-headed arrow). Notice that metallic applicator mandrins have been replaced by non-visible laser fibers.

Temperature-sensitive T1-weighted imaging revealed an almost linear signal decrease with rising temperature in the ablation zone (p_5_ = 0.068; p_10_ = 1.000; p_15_ = 0.753; p_20_ = 0.109). (see figs. 6 and 7) CNR concordantly increased with duration of therapy and rising temperature in the target zone but did not significantly differ between both groups one and two (p_5_ = 0.144; p_10_ = 0.465; p_15_ = 0.463; p_20_ = 0.593)

## Discussion

Intravenous employment of Gd-EOB-DTPA for periinterventional contrast enhancement in MR-guided diagnostic liver interventions has been reported in the literature [24], [25]. Fischbach et al. were able to show that a T1-weighted spoiled gradient echo sequence (T1FFE) is suitable and non-inferior to another diagnostic GRE sequence for Gd-EOB-DTPA-enhanced real-time guidance in their setting for diagnostic liver biopsy, focusing on instrument guidance in a 1.0 T open MR scanner [25]. Our study instead comprises a high-field closed bore setting at 1.5 T utilizing repetitive acquisition of fast GRE sequences for applicator guidance. The procedural setting is primarily determined by the need of high spatial resolution, for the intended laser ablation is being controlled through real-time MR thermometry within the target zone. We are aware of a case report emphasizing promotive effects of additional Gd-EOB-DTPA when performing MR-guided RFA, but the report lacks any systematic work-up [24]. Even though the beneficial nature of “whitening” the liver for about an hour seams obvious when performing MR-guided interventions, which are based on metallic instruments targeting “blackened” focal liver lesions, circumstances have rarely been proven in reliable tests. Particularly when performing therapeutic laser ablation with modality-specific parallel placement of multiple applicators per procedure, beneficial effects are equally expected to multiply. Inter-individual differences in responding the promotive measure are widely unknown. First of all our results confirm the appropriateness of fast T1-weighted FLASH 3D GRE sequences to both utilize contrast-induced imaging effects and being sufficient to display the instrument during placement period in a 3D-reconstructive manor. Inter-object signal modifications within the target organ liver were diverse and are discussed in the following sections.

### Contrast-induced tissue signal alteration in the target organ

As expected from daily diagnostic practice and as known from the literature [23] bolus application of gadoxetic acid in the given manor was sufficient to homogeneously increase signal intensity in healthy liver parenchyma, also in our study setting. Signal elevation was statistically significant and reliably lasted for the whole procedure monitoring up to 60 minutes. Signal changes started with a rapid increase reaching maximum values after 10–15 minutes, before entering a plateau-like progression within the residual catheter placement phase (see fig. 1). These findings correlate with results from previous international studies to which the Munich study group on Gd-EOB-DTPA contributed most [20], [22], [31]–[34]. Reported signal peaks vary between 10 and 20 minutes after administration of the contrast agent, representing the cellular uptake phase. Differences in the timeline may be partially due to the study design using 5-minute corridors to monitor signal alteration. A finer temporal granulation of surveillance was not seen necessary according to the sequential nature of the applicator placement process.

The targeted tumors within the livers represented the second focus of surveillance. Appropriate dynamic contrast media imaging in terms of diagnostic early phase work-up was not part of the study design. Even though, post-contrast image acquisition started at different time points within the period of one minute after injection and therefore also monitored the early vascular phase of intravenous Gd-EOB-DTPA. Contrast behavior in all focal metastatic or neoplastic liver lesions was concordant with the literature [22], [33], [35]. One case of focal secondary HCC presented the highest peak of early arterial phase enhancement doubling the initial signal intensity, highly consistent with its hypervascular properties. Secondary HCC in a different individual, at the same time, showed no contrast enhancement at all after i.v. bolus. Histologic proof in this case had not been found critical, as tumors were adequately diagnosed recurrent disease. In consequence, histologic work-up could not reveal a possible regressive nature of the target tumor. All other hepatic tumors were metastases of different alien origin. Early vascular uptake in these tumors was present and peaked within the first 5 minutes for the vast majority of cases. At 25 minutes for the latest, SNR values for all target tumors, HCC and metastases, had entered an attenuation steady state, consistent with dedifferentiated or non-hepatic tissue.

### Ensuing contrast-induced periprocedural imaging effects

With both a contrast-induced lasting signal increase in the liver parenchyma and an attenuation steady state in targeted tumors during late phase, calculation of the actual contrasting effect (CNR) between tissues and/or instrumentation was a major interest of the study. Evaluation results strongly support the hypothesis of a promotive effect for Gd-EOB-DTPA when it comes to periinterventional delineation and differentiation of the three corresponding imaging targets – applicator, tumor and liver parenchyma.

Monitoring CNR (representing the delta in signal intensity) for target tumors which did not take up gadoxetic acid at any time, revealed an immediate and exclusively positive curve progression, representing an all-time high-low contrast between liver and tumor, with maximum contrast values at 12 to 18 minutes after administration of Gd-EOB-DTPA. CNR changes were highly significant. When monitoring all other tumors, early enhancing exceeded that of liver parenchyma and led to a negative curve progression with maximum CNR values below zero after almost 5 minutes, representing a transient low-high contrast between liver and tumor. Trespassing an equilibrium phase CNR curves turned positive after an average ¼ of an hour and progressed to maximum contrast values at less than ½ an hour. Reimer et al. [36] presented a similar biphasic curve progression for enhancing target tumors but found corresponding peak negative contrast after less than two minutes, followed by a phase of positive contrast beginning as early as three minutes after i.v. injection of gadoxetic acid. Findings for late phase contrasting maxima in our study are scattered around an average 20 minutes known from the literature [32], [36]. Diagnostically advantageous delineation of even smallest focal liver malignancies, that made contrast-enhanced MRI today's gold standard in imaging focal liver lesions [37], offers the same beneficial properties when it comes to MR-guided invasive instrumentation that is targeting those focal malignancies. More than that, abiding the cell-specific late phase in this case is not a necessary evil to gain diagnostic information but, instead, most favorable to support the intervention lasting between 10 and 60 minutes. Converse contrast behavior within the first 5 minutes after medication, as shown by a number of targeted tumors, is neglectable in the given setting, where most of the guiding process is performed after this period, especially when using multiple applicators.

Fischbach et al. [25] reported an improved tracking of their titanium needle from 20 to 40 minutes after application of gadoxetic acid, when performing open bore MR-guided punch biopsy in the liver. Our findings show promotive contrasting maxima, representing a high-low liver-to-applicator contrast, less than 15 minutes after administration of gadoxetic acid and not fading until catheter placement was finished (up to 60 minutes). Even contrasting effects between applicator and punctured tumor were significantly superior to those of the non-enhanced control group. This correlated with maintained residual elevation of signal intensity in target tumor tissue even after more than 40 minutes. After all, monitoring and quantifying of inter-tissue contrasting effects over time in the given study revealed significant improvement for the contrast-enhanced group at three tracking points – the target tumor being silhouetted against the liver, the manually advanced applicator being silhouetted against the liver and the applicator being silhouetted against the speared tumor. Every one of the three tracking elements on its own is beneficial to the visualization process at the human-screen interface and therefore critical for an efficient workflow in the given medical task. In addition, the convenient time course of the medication-induced promotive impact has been proven suitable for percutaneous MR-guided tumor targeting, in particular when performing a sequential multi-needle procedure.

### Contrast influence on therapy monitoring

Results of this study showed that the use of gadoxetic acid does not interfere with T1 thermometric imaging, which is used to monitor the thermal ablation procedure [9]. The therapy monitoring phase is differentiated from the catheter placement phase. At this time the metallic applicator has been replaced by a flexible laser fiber, which per se is not visible on the MR images. Besides exclusion of unintended side effects, such as bleeding, online therapy monitoring utilizes temperature mapping to judge on sufficient therapy impact on the one hand and to avoid harming temperature-sensitive neighboring structures on the other hand [38], [39]. T1-based MRI thermometry for extrinsic oncologic therapy control represents a high-end imaging protocol for thermal tumor ablation [7], [38]. It relies on high-resolution imaging in the target zone and therefore is a major criterion to perform in a high-field closed bore system. Consistent with earlier studies [9], [40] we found a continuing, almost linear decrease in T_1_-weighted signal within the target zone and growing from the active tip of the laser fiber. Signal drop and zone growing both are saturable, finalizing in an asymptotic approximation to minimum or maximum values, usually explained with limited penetration depth and heat-induced tissue alteration [38]. The liver-to-impact contrast analogously increased for at least 15 of 20 minutes total ablation time. These results were identical in both groups, being monitored, with comparative analysis not showing any statistic significance.

In conclusion results strongly support the anticipated promotive effect of Gd-EOB-DTPA for MR-guided percutaneous liver interventions by quantifying the delineating effects for therapy-relevant elements in the procedure. Even though interindividual differences could be measured for timing and degree of the promotive effects in a small patient cohort, study results impressively verify prevailing visual advantages through the use of Gd-EOB-DTPA, without respect to tumor entity or undefined individual metabolic factors. As a preliminary measure and based on the results of this study the reporting group performs MR-guided liver interventions only after Gd-EOB-DTPA enhancement, if not contraindicated. Time benefit, cost effectiveness and oncologic outcome of the described beneficiary effects will have to be part of further investigations.

## References

[1] Gillams AR, Lees WR (2005) Radiofrequency ablation of colorectal liver metastases. Abdom Imaging 30:419–426. 15759208 10.1007/s00261-004-0256-6

[2] Lencioni R, Crocetti L (2013) Image-guided ablation for hepatocellular carcinoma. Recent Results Cancer Res 190:181–194. 22941021 10.1007/978-3-642-16037-0_12

[3] Mahnken AH, Pereira PL, de Baere T (2013) Interventional oncologic approaches to liver metastases. Radiology 266:407–430. 23362094 10.1148/radiol.12112544

[4] McDermott S, Gervais DA (2013) Radiofrequency Ablation of Liver Tumors. Semin Intervent Radiol 30:49–55. 24436517 10.1055/s-0033-1333653 PMC3700792

[5] Nour SG, Lewin JS (2005) Radiofrequency thermal ablation: the role of MR imaging in guiding and monitoring tumor therapy. Magn Reson Imaging Clin N Am 13:561–581. 16084420 10.1016/j.mric.2005.04.007

[6] de Senneville BD, Mougenot C, Quesson B, Dragonu I, Grenier N, et al (2007) MR thermometry for monitoring tumor ablation. Eur Radiol 17:2401–2410. 17701184 10.1007/s00330-007-0646-6

[7] Kickhefel A, Rosenberg C, Weiss CR, Rempp H, Roland J, et al (2011) Clinical evaluation of MR temperature monitoring of laser-induced thermotherapy in human liver using the proton-resonance-frequency method and predictive models of cell death. J Magn Reson Imaging 33:704–712. 21563256 10.1002/jmri.22499

[8] Lepetit-Coiffe M, Laumonier H, Seror O, Quesson B, Sesay MB, et al (2010) Real-time monitoring of radiofrequency ablation of liver tumors using thermal-dose calculation by MR temperature imaging: initial results in nine patients, including follow-up. Eur Radiol 20:193–201. 19657650 10.1007/s00330-009-1532-1

[9] Puls R, Langner S, Rosenberg C, Hegenscheid K, Kuehn JP, et al (2009) Laser ablation of liver metastases from colorectal cancer with MR thermometry: 5-year survival. J Vasc Interv Radiol 20:225–234. 19109037 10.1016/j.jvir.2008.10.018

[10] Rempp H, Clasen S, Boss A, Roland J, Kickhefel A, et al (2009) Prediction of cell necrosis with sequential temperature mapping after radiofrequency ablation. J Magn Reson Imaging 30:631–639. 19630076 10.1002/jmri.21863

[11] Vogl TJ, Straub R, Zangos S, Mack MG, Eichler K (2004) MR-guided laser-induced thermotherapy (LITT) of liver tumours: experimental and clinical data. Int J Hyperthermia 20:713–724. 15675667 10.1080/02656730400007212

[12] Crocetti L, de Baere T, Lencioni R (2010) Quality improvement guidelines for radiofrequency ablation of liver tumours. Cardiovasc Intervent Radiol 33:11–17. 19924474 10.1007/s00270-009-9736-y PMC2816824

[13] Goldberg SN, Grassi CJ, Cardella JF, Charboneau JW, Dodd GD 3rd, et al (2009) Image-guided tumor ablation: standardization of terminology and reporting criteria. J Vasc Interv Radiol 20:377–390. 10.1097/01.RVI.0000170858.46668.65 15947040

[14] Rosenberg C, Kickhefel A, Mensel B, Pickartz T, Puls R, et al (2013) PRFS-based MR thermometry versus an alternative T1 magnitude method–comparative performance predicting thermally induced necrosis in hepatic tumor ablation. PLoS One 8:e78559. 24205260 10.1371/journal.pone.0078559 PMC3813475

[15] Stafford RJ, Fuentes D, Elliott AA, Weinberg JS, Ahrar K (2010) Laser-induced thermal therapy for tumor ablation. Crit Rev Biomed Eng 38:79–100. 21175405 10.1615/critrevbiomedeng.v38.i1.70

[16] Kuhn FP, Crook DW, Mader CE, Appenzeller P, von Schulthess GK, et al (2013) Discrimination and anatomical mapping of PET-positive lesions: comparison of CT attenuation-corrected PET images with coregistered MR and CT images in the abdomen. Eur J Nucl Med Mol Imaging 40:44–51. 22955547 10.1007/s00259-012-2236-3

[17] Puls R, Stroszczynski C, Rosenberg C, Kuehn JP, Hegenscheid K, et al (2007) Three-dimensional gradient-echo imaging for percutaneous MR-guided laser therapy of liver metastasis. J Magn Reson Imaging 25:1174–1178. 17520737 10.1002/jmri.20936

[18] Ba-Ssalamah A, Uffmann M, Saini S, Bastati N, Herold C, et al (2009) Clinical value of MRI liver-specific contrast agents: a tailored examination for a confident non-invasive diagnosis of focal liver lesions. Eur Radiol 19:342–357. 18810454 10.1007/s00330-008-1172-x

[19] Bluemke DA, Sahani D, Amendola M, Balzer T, Breuer J, et al (2005) Efficacy and safety of MR imaging with liver-specific contrast agent: U.S. multicenter phase III study. Radiology 237:89–98. 16126918 10.1148/radiol.2371031842

[20] Hamm B, Staks T, Muhler A, Bollow M, Taupitz M, et al (1995) Phase I clinical evaluation of Gd-EOB-DTPA as a hepatobiliary MR contrast agent: safety, pharmacokinetics, and MR imaging. Radiology 195:785–792. 7754011 10.1148/radiology.195.3.7754011

[21] Hammerstingl R, Huppertz A, Breuer J, Balzer T, Blakeborough A, et al (2008) Diagnostic efficacy of gadoxetic acid (Primovist)-enhanced MRI and spiral CT for a therapeutic strategy: comparison with intraoperative and histopathologic findings in focal liver lesions. Eur Radiol 18:457–467. 18058107 10.1007/s00330-007-0716-9

[22] Raman SS, Leary C, Bluemke DA, Amendola M, Sahani D, et al (2010) Improved characterization of focal liver lesions with liver-specific gadoxetic acid disodium-enhanced magnetic resonance imaging: a multicenter phase 3 clinical trial. J Comput Assist Tomogr 34:163–172. 20351497 10.1097/RCT.0b013e3181c89d87 PMC3036163

[23] Seale MK, Catalano OA, Saini S, Hahn PF, Sahani DV (2009) Hepatobiliary-specific MR contrast agents: role in imaging the liver and biliary tree. Radiographics 29:1725–1748. 19959518 10.1148/rg.296095515

[24] Bathe OF, Mahallati H (2007) MR-guided ablation of hepatocellular carcinoma aided by gadoxetic acid. J Surg Oncol 95:670–673. 17345591 10.1002/jso.20768

[25] Fischbach F, Thormann M, Seidensticker M, Kropf S, Pech M, et al (2011) Assessment of fast dynamic imaging and the use of Gd-EOB-DTPA for MR-guided liver interventions. J Magn Reson Imaging 34:874–879. 21769983 10.1002/jmri.22691

[26] Vander Elst L, Maton F, Laurent S, Seghi F, Chapelle F, et al (1997) A multinuclear MR study of Gd-EOB-DTPA: comprehensive preclinical characterization of an organ specific MRI contrast agent. Magn Reson Med 38:604–614. 9324328 10.1002/mrm.1910380415

[27] Sieber MA, Steger-Hartmann T, Lengsfeld P, Pietsch H (2009) Gadolinium-based contrast agents and NSF: evidence from animal experience. J Magn Reson Imaging 30:1268–1276. 19938039 10.1002/jmri.21971

[28] Parker DL (1984) Applications of NMR imaging in hyperthermia: an evaluation of the potential for localized tissue heating and noninvasive temperature monitoring. IEEE Trans Biomed Eng 31:161–167. 6724602 10.1109/TBME.1984.325382

[29] Fahrmeir L, Brachinger W, Hamerle A, Tutz G (1996) Multivariate statistische Verfahren. Berlin, New York: de Gruyter. 902 p.

[30] Wolf C, Best H (2010) Handbuch der sozialwissenschaftlichen Datenanalyse. Wiesbaden: VS-Verlag. 1098 p.

[31] Filippone A, Blakeborough A, Breuer J, Grazioli L, Gschwend S, et al (2010) Enhancement of liver parenchyma after injection of hepatocyte-specific MRI contrast media: a comparison of gadoxetic acid and gadobenate dimeglumine. J Magn Reson Imaging 31:356–364. 20099349 10.1002/jmri.22054

[32] Frericks BB, Loddenkemper C, Huppertz A, Valdeig S, Stroux A, et al (2009) Qualitative and quantitative evaluation of hepatocellular carcinoma and cirrhotic liver enhancement using Gd-EOB-DTPA. AJR Am J Roentgenol 193:1053–1060. 19770329 10.2214/AJR.08.1946

[33] Halavaara J, Breuer J, Ayuso C, Balzer T, Bellin MF, et al (2006) Liver tumor characterization: comparison between liver-specific gadoxetic acid disodium-enhanced MRI and biphasic CT–a multicenter trial. J Comput Assist Tomogr 30:345–354. 16778605 10.1097/00004728-200605000-00001

[34] Reimer P, Rummeny EJ, Shamsi K, Balzer T, Daldrup HE, et al (1996) Phase II clinical evaluation of Gd-EOB-DTPA: dose, safety aspects, and pulse sequence. Radiology 199:177–183. 8633143 10.1148/radiology.199.1.8633143

[35] Lencioni R, Crocetti L, Della Pina MC, Cioni D (2008) Guidelines for imaging focal lesions in liver cirrhosis. Expert Rev Gastroenterol Hepatol 2:697–703. 19072346 10.1586/17474124.2.5.697

[36] Reimer P, Rummeny EJ, Daldrup HE, Hesse T, Balzer T, et al (1997) Enhancement characteristics of liver metastases, hepatocellular carcinomas, and hemangiomas with Gd-EOB-DTPA: preliminary results with dynamic MR imaging. Eur Radiol 7:275–280. 9038130 10.1007/s003300050150

[37] Schmiegel W, Pox C, Reinacher-Schick A, Adler G, Arnold D, et al (2010) S3 guidelines for colorectal carcinoma: results of an evidence-based consensus conference on February 6/7, 2004 and June 8/9, 2007 (for the topics IV, VI and VII). Z Gastroenterol 48:65–136. 20072998 10.1055/s-0028-1109936

[38] Rieke V, Butts Pauly K (2008) MR thermometry. J Magn Reson Imaging 27:376–390. 18219673 10.1002/jmri.21265 PMC2780364

[39] Vogl TJ, Weinhold N, Mack MG, Muller PK, Scholz WR, et al (1998) Verification of MR thermometry by means of an in vivo intralesional, fluoroptic temperature measurement for laser-induced thermotherapy ov liver metastases. Fortschr Roentgenstr 169:182–188. 10.1055/s-2007-1015071 9739370

[40] Meister D, Hubner F, Mack M, Vogl TJ (2007) MR thermometry for laser-induced thermotherapy at 1.5 Tesla. Fortschr Roentgenstr 179:497–505. 10.1055/s-2007-962979 17436184

